# Novel features on the regulation by mitochondria of calcium and secretion transients in chromaffin cells challenged with acetylcholine at 37°C

**DOI:** 10.1002/phy2.182

**Published:** 2013-12-19

**Authors:** Afonso Caricati‐Neto, Juan‐Fernando Padín, Edilson‐Dantas Silva‐Junior, José‐Carlos Fernández‐Morales, Antonio‐Miguel G. de Diego, Aron Jurkiewicz, Antonio G. García

**Affiliations:** 1 Departamento de Farmacología, Escola Paulista de Medicina, Universidade Federal de São Paulo, São Paulo, Brazil; 2 Instituto Teófilo Hernando, Facultad de Medicina, Universidad Autónoma de Madrid, C/Arzobispo Morcillo, 4, Madrid, 28029, Spain; 3 Departamento de Farmacología y Terapéutica, Facultad de Medicina, Universidad Autónoma de Madrid, C/Arzobispo Morcillo, 4, 28029Madrid, Spain; 4 Servicio de Farmacología Clínica, Instituto de Investigación Sanitaria, Hospital Universitario de la Princesa, Universidad Autónoma de Madrid, C/Diego de León, 62, Madrid, 28006, Spain

**Keywords:** Calcium homeostasis, catecholamines, CGP37157, chromaffin cells, exocytosis, FCCP, mitochondria, oligomycin, rotenone, Ru360

## Abstract

From experiments performed at room temperature, we know that the buffering of Ca^2+^ by mitochondria contributes to the shaping of the bulk cytosolic calcium transient ([Ca^2+^]_c_) and secretion transients of chromaffin cells stimulated with depolarizing pulses. We also know that the mitochondrial Ca^2+^ transporters and the release of catecholamine are faster at 37°C with respect to room temperature. Therefore, we planned this investigation to gain further insight into the contribution of mitochondrial Ca^2+^ buffering to the shaping of [Ca^2+^]_c_ and catecholamine release transients, using some novel experimental conditions that have not been yet explored namely: (1) perifusion of bovine chromaffin cells (BCCs) with saline at 37°C and their repeated challenging with the physiological neurotransmitter acetylcholine (ACh); (2) separate blockade of mitochondrial Ca^2+^ uniporter (mCUP) with Ru360 or the mitochondrial Na^+^/Ca^2+^ exchanger (mNCX) with CGP37157; (3) full blockade of the mitochondrial Ca^2+^ cycling (mCC) by the simultaneous inhibition of the mCUP and the mNCX. Ru360 caused a pronounced delay of [Ca^2+^]_c_ clearance and augmented secretion. In contrast, CGP37157 only caused a tiny delay of [Ca^2+^]_c_ clearance and a mild decrease in secretion. The mCC resulting in continued Ca^2+^ uptake and its release back into the cytosol was interrupted by combined Ru360 + CGP37157 (Ru/CGP), the protonophore carbonyl cyanide‐p‐trifluoromethoxyphenylhydrazone, or combined oligomycin + rotenone (O/R); these three treatments caused a mild but sustained elevation of basal [Ca^2+^]_c_ that, however, was not accompanied by a parallel increase in basal secretion. Nevertheless, all treatments caused a pronounced augmentation of ACh‐induced secretion, with minor changes of the ACh‐induced [Ca^2+^]_c_ transients. Combined Ru/CGP did not alter the resting membrane potential in current‐clamped cells. Additionally, Ru/CGP did not increase basal [Ca^2+^]_c_ near subplasmalemmal sites and caused a mild decrease in the size of the readily releasable vesicle pool. Our results provide new functional features in support of the view that in BCCs there are two subpopulations of mitochondria, M1 underneath the plasmalemma nearby exocytotic sites and M2 at the core cell nearby vesicle transport sites. While M1 serves to shape the ACh‐elicited exocytotic response through its efficient Ca^2+^ removal by the mCUP, M2 shapes the lower [Ca^2+^]_c_ elevations required for new vesicle supply to the exocytotic machinery, from the large reserve vesicle pool at the cell core. The mCUP of the M1 pool seems to play a more prominent role in controlling the ACh responses, in comparison with the mNCX.

## Abstract (precis)

Regulation by mitochondria of exocytosis at 37°C.

## Introduction

Mitochondria are the main energy‐producing centers of eukaryotic cells (Duchen 2000). During cell activation, they are capable of accumulating in their matrix vast amounts of Ca^2+^ through their mitochondrial Ca^2+^ uniporter (mCUP) that uses the driving force of the electrical potential across the mitochondrial membrane (Reynafarje and Lehninger 1977). After cell stimulation ceases, the Ca^2+^ accumulated in the mitochondrial matrix is then released back into the cytosol by electroneutral antiporters that export Ca^2+^ from the matrix by swapping one Ca^2+^ ion for Na^+^ through the mitochondrial Na^+^/Ca^2+^ exchanger (mNCX) (Carafoli 1979; Gunter and Pfeiffer 1990). This mitochondrial Ca^2+^ cycling (mCC) was first shown to occur in isolated cardiac mitochondria (Crompton and Heid 1978). Later on, it was also demonstrated that N‐methyl‐D‐aspartate receptor (NMDA)‐induced Ca^2+^ loads recycle across the inner mitochondrial membrane of hippocampal neurons in culture (Wang and Thayer 2002).

In chromaffin cells (CCs) from the adrenal gland, mitochondria play an essential role in clearing the cytosolic calcium transient ([Ca^2+^]_c_) transient generated by depolarizing stimuli, both in rats (Herrington et al. 1996; Babcock et al. 1997) and calves (Montero et al. 2000; Villalobos et al. 2002). In bovine CCs (BCCs) transfected with mitochondrial‐targeted aequorins with low affinity for Ca^2+^, we measured a maximal mitochondrial Ca^2+^ uptake as high as 6 mmol · L cells^−1^ · s^−1^ (Villalobos et al. 2002). During maximal stimulation of Ca^2+^ entry by depolarization with high K^+^ of BCCs, mitochondria took up about 1.1 mmol · L cells^−1^ · s^−1^, a value comparable with the rate of Ca^2+^ entry through voltage‐activated Ca^2+^ channels (VACCs) (Montero et al. 2000; Villalobos et al. 2002). Most Ca^2+^ release from Ca^2+^‐loaded mitochondria occurs through the mNCX at the rate of 0.8 mmol · L cells^−1^ · s^−1^ at 37°C. The sensitivity of this system to temperature is very high and, at room temperature (22–24°C), Ca^2+^ efflux through the mNCX is considerably slower (Montero et al. 2000).

This mCC surely has physiological relevance because the dissipation of the proton gradient by protonophores carbonyl cyanide‐p‐trifluoromethoxyphenylhydrazone (FCCP) or carbonyl cyanide m‐chlorophenyl hydrazone (CCCP) suppresses the Ca^2+^ buffering capacity of mitochondria and causes an increase in exocytosis in voltage‐clamped BCCs (Giovannucci et al. 1999). On the other hand, in perifused BCCs stimulated with acetylcholine (ACh), caffeine, or K^+^, the protonophores also cause augmentation of catecholamine release (Montero et al. 2000, 2002; Cuchillo‐Ibanez et al. 2004). This potentiation of secretion also occurs in rat CCs (Miranda‐Ferreira et al. 2009). Surprisingly, in mouse CCs protonophores actually reduce the K^+^‐elicited [Ca^2+^]_c_ transients and secretory responses (Ales et al. 2005); this can be explained by differences in the expression of VACCs subtypes in bovine versus mouse CCs (Garcia et al. 2006) and/or by different rates and extent of inactivation of those channel types during suppression by protonophores of mitochondrial Ca^2+^ buffering (Hernandez‐Guijo et al. 2001).

The information obtained with the experiments just mentioned is certainly useful. However, from a physiological perspective the knowledge generated is of limited value, for the following reasons: (1) experiments previously referenced have been carried out at room temperature; (2) mitochondrial Ca^2+^ transport is highly sensitive to temperature and for instance, the rate of Ca^2+^ efflux through the mNCX is accelerated fivefold at 37°C with respect to 22°C (Montero et al. 2000; Villalobos et al. 2002); (3) the exocytotic release of catecholamine also exhibits a high temperature dependence (Knight and Baker 1983; Kao and Westhead 1984; Bittner and Holz 1992; Padin et al. 2013); (4) only drastic treatments with protonophores to suppress the mNCX (Ca^2+^ uptake and release) were previously used; (5) only in one study (Montero et al. 2000) cells were challenged with the physiological neurotransmitter ACh (Feldberg et al. 1934).

In this study we planned a more physiological approach to the understanding of the role of the mCC to shape the [Ca^2+^]_c_ transients and catecholamine release response, generated by repeated challenging with ACh of fast‐perifused BCCs with a saline solution at 37°C, using some new pharmacological tools not yet used in this system as well as already used tools namely: (1) Ru360, a mCUP blocker with cell permeability higher than ruthenium red (Zazueta et al. 1999); (2) CGP37157 to decrease the rate of mitochondrial Ca^2+^ efflux through the mNCX (Cox et al. 1993); (3) Combined use of Ru360 and CGP37157 (Ru/CGP) to decrease the rate of the mNCX, that is, the continued Ca^2+^ uptake/release occurring in mitochondria during cell activation and after the stimulus (Montero et al. 2000; Villalobos et al. 2002); (4) FCCP to suppress the proton gradient and stop the mitochondrial Ca^2+^ uptake (Montero et al. 2000); and (5) Combined use of oligomycin (to block ATP synthase) and rotenone (to block Complex I of the respiratory chain) (O/R), to also decrease the mNCX. The two novel main findings of this study are as follows: First, the high‐capacity mCUP of a subpopulation M1 of mitochondria underneath the plasmalemma rapidly clear the [Ca^2+^]_c_ transient to regulate the extent and time course of the exocytotic response elicited by ACh at 37°C; and second, the mCC of a M2 subpopulation of mitochondria at inner cytosolic sites works between ACh pulses to shape the small but sustained [Ca^2+^]_c_ elevations to favor the transport of new vesicles to the exocytotic machinery.

## Material and Methods

### Ethical approval

Bovine adrenal glands were obtained from a local slaughterhouse that legally and ethically sacrificed the calves for human feeding. Thus, in this case the ethical issues on the use of animals for laboratory experiments, in accordance with the Declaration of Helsinki and the approval of the Ethical Committee for the care and use of animals, of the Medical School, Autonomous University of Madrid, Spain, in accordance with the Directive 2010/63/EU of the European Parliament and of the Council of 22 September 2010 on the protection of animals used for scientific purposes and with the Spanish Real Decreto of 10 October 2005 (RD 1201/2005), does not apply.

### Isolation and culture of bovine adrenal CCs

BCCs were isolated from adrenal glands of calves (*Bos taurus*), according to standard methods (Livett 1984) with some modifications (Moro et al. 1990). Cells were suspended in Dulbecco's modified Eagle's medium (DMEM) supplemented with 5% fetal calf serum, 10 *μ*mol/L cytosine arabinoside, 10 *μ*mol/L fluorodeoxyuridine, 50 IU/mL penicillin, and 50 *μ*g/mL streptomycin. For measurements of catecholamine secretion from superfused populations of BCCs, cells were plated on 60‐mm diameter plastic Petri dishes at a density of 10^6^ cells/mL (5 mL/dish). To measure ACh‐stimulated [Ca^2+^]_c_ elevations in BCCs loaded with fura‐2 or FFP18, cells were plated at a density of 5 × 10^5^ cells/coverslip in 25‐mm 6‐well plates. Cells were kept for 2–4 days in a water‐saturated incubator at 37°C, in a 5% CO_2_‐95% air atmosphere, and used 1–4 days thereafter. After 24 h, the medium was replaced by serum‐free fresh medium and subsequently changed every 2 days.

### On‐line amperometric recordings of burst catecholamine release from superfused populations of BCCs

An online amperometric method was used to measure at real time the rate of ACh‐stimulated catecholamine release from BCCs populations (Borges et al. 1986). Around five million cells per experiment were scrapped off carefully from the bottom of the Petri dish with a rubber policeman, and centrifuged at 200 g (Heraeus centrifuge, Madrid, Spain) for 10 min. The cell pellet was resuspended in 200 *μ*L of Krebs–HEPES (composition in mmol/L: 144 NaCl, 5.9 KCl, 1.2 MgCl_2_, 2 CaCl_2_, 11 glucose, and 10 HEPES, pH 7.4). Cells were introduced in a 100 *μ*L‐microchamber for their superfusion at the constant rate of 2 mL/min. The liquid flowing from the superfusion chamber reached an electrochemical detector model 641 VA (Metrohm, Herisau, Switzerland), placed just at the outlet of the microchamber, which monitors online the amount of catecholamines secreted under the amperometric mode. Cells were stimulated to secrete with short pulses (5 sec) of a Krebs–HEPES solution containing 30 *μ*mol/L ACh (concentration corresponding to EC_50_ of ACh to produce a secretory response in BCCs). Solutions were rapidly exchanged through electrovalves commanded by a computer. This amperometric strategy permits the online recording of reproducible catecholamine release responses during long time periods of 30–60 min. In this study, all experiments were performed at the constant temperature of 37 ± 1°C. The maximal amperometric signal stimulated with ACh corresponding to secretion peak (SP) was evaluated. To estimate the quantity of catecholamine secreted per pulse, the signal expressed in nano ampere (nA) corresponding to SP was compared to peak of amperometric signal obtained with different concentrations of adrenaline (1–20 *μ*mol/L). An analysis of amperometric charge of SP was calculated by integrating the amperometric current over time during the stimulus duration by means of Origin Pro 8 SR2 software, version 8.0891 (OriginLab Corporation, Northampton, MA).

### Measurements of changes of [Ca^2+^]_c_ in single BCCs

At the moment of Ca^2+^ measurements, BCCs were incubated for 1 h at 37°C in DMEM containing the Ca^2+^ probe Fura‐2 AM (10 *μ*mol/L) or the near‐membrane Ca^2+^ indicator FFP18 (Etter et al. 1996). After this incubation period, the coverslips were mounted in a chamber, and cells were washed and covered with Tyrode solution composed of (mmol/L): 137 NaCl, 1 MgCl_2_, 5.3 KCl, 2 CaCl_2_, 10 HEPES, and 10 glucose, pH 7.4 with NaOH. The setup for fluorescence recordings was composed of a Leica DMI 4000 B inverted light microscope (Leica Microsystems, Barcelona, Spain) equipped with an oil immersion objective (Leica 40 × Plan Apo; numerical aperture 1.25). Once the cells were placed under the microscope, they were continuously superfused by means of a five‐way superfusion system at 1 mL/min with a common outlet 0.28 mm‐tube driven by electrically controlled valves with Tyrode's solution, at 37°C. Fura‐2 was excited alternatively at 340 ± 10 nm and 387 ± 10 nm using a Küber CODIX xenon 8 lamp (Leica, Barcelona, Spain). Emitted fluorescence was collected through a 540 ± 20 nm emission filter and measured with an intensified charge coupled device camera (Hamamatsu camera controller C10600 orca R2; Barcelona, Spain). Fluorescence images were generated at 1‐sec intervals. Images were digitally stored and analyzed using LAS AF software (Leica, Barcelona, Spain). A Tyrode external solution was used of the same composition as the one described above, where drugs were added to this solution. The rates of exponential decay of ACh‐induced [Ca^2+^]_c_ responses were compared between BCCs treated and untreated with agents, which interfere with Ca^2+^ transport by mitochondria.

### Recording of whole‐cell perforated patch‐clamp Em and the ΔCm

Resting membrane potential (Em), capacitance increments (ΔCm), and calcium currents were recorded using standard patch‐clamp techniques with an EPC‐10 amplifier and PULSE software (HEKA‐elektronics, Lambrecht/Pfalz, Germany) on the stage of a Nikon Diaphot microscope equipped with Narishige MWH‐3 micromanipulators (London, U.K.), as previously described (de Diego et al. 2008). All recordings were carried out using the perforated‐patch mode. The bath solution for all recording was composed of (in mmol/L): 137 NaCl, 1 MgCl_2_, 2 CaCl_2_, 5 KCl, 10 HEPES, and 10 glucose, pH 7.35 with NaOH.

We used two intracellular solutions; solution 1 for current clamp recordings to measure membrane potential variations was composed of (in mmol/L): 135 KCl, 9 NaCl, 10 HEPES, 1 MgCl_2_; pH was titrated with KOH to 7.2. Solution 2 for the recording of ΔCm and calcium currents under voltage clamp contained (in mmol/L): 145 glutamic acid, 1 MgCl_2_, 8 NaCl, and 10 HEPES, pH 7.2 with CsOH. Leak currents in voltage‐clamp were automatically subtracted by the PULSE program by applying a Pn/P4 hyperpolarizing protocol.

### Size estimation of ready‐release vesicle pool (RRVP) in single BCCs

To estimate the size of the RRVP, two 100‐msec depolarizing pulses were given 100 msec apart in the perforated‐patch configuration and 2 mmol/L extracellular Ca^2+^. Depolarizations were adjusted to match calcium current sizes of both pulses (Gillis et al. 1996). ΔCm was calculated by subtracting the mean of a 20 msec capacitance window just after 25 msec of the end of the depolarizing pulse. We calculated the parameters *B*_min_ and *B*_max_ as previously described (Gillis et al. 1996), which produce a minimum and maximum value for the size of a given vesicle pool.

### Chemical products

The following chemicals were used for the bovine cell culture: DMEM, gentamicin and penicillin‐streptomycin were from GIBCO (Scotland, U.K.), l‐glutamine was from Sigma (Sigma‐Aldrich, Madrid, Spain), fetal bovine serum was from PAA laboratories (Pasching, Austria), and collagenase‐A from *Clostridium histolyticum* was from Roche diagnostics GmbH (Mannheim, Germany). The probes Fura‐2 AM and FFP18 were supplied by Invitrogen (Eugene, OR). FCCP, oligomycin, and rotenone were obtained from Sigma (Sigma‐Aldrich, Madrid, Spain). CGP37157 was obtained from Tocris (Bristol, U.K.) and Ru‐360 from Calbiochem (Philadelphia, PA).

### Data analysis and statistics

Regarding cytosolic Ca^2+^ concentrations data analysis was carried out on a personal computer; data obtained from LAS AF Lite software, version 2.6.0 (Barcelona, Spain) were exported to Excel tables (Microsoft, Redmond, WA). Graphs and the mathematical analyses were performed using the Graphpad Prism software, version 5.01 (GraphPad Software Inc, San Diego, CA). Areas or peaks heights were calculated by integrating the calcium transient over time during the stimulus duration by means of Origin Pro 8 SR2 software, version 8.0891 (OriginLab Corporation, Northampton). Results shown in the text and figures are expressed as mean ± SEM. Unless otherwise stated, statistical analyses were carried out with analysis of variance (ANOVA) one‐way test, and Tukey post hoc analyses; *, **, or *** show a statistical significance of *P *< 0.05, *P *< 0.01 or *P *< 0.001, respectively. Kinetic parameters of [Ca^2+^]_c_ transients were determined using Origin Pro 8 SR2 software, version 8.0891 (OriginLab Corporation, Northampton). The [Ca^2+^]_c_ transients were fitted to the exponential decay equation
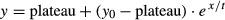
1where *x* is the time, *y* starts at *y*_0_ and decays down or goes up to plateau, and *t* is the time or rate constant equal to the reciprocal of the *x* axis. The kinetic data of catecholamine release transients were calculated using a similar approach.

## Results

### The [Ca^2+^]_c_ and secretory transients elicited by repeated pulses of ACh

In this study, care was taken to perform the experiments under conditions close to physiology. Thus, the stimulus was ACh, the neurotransmitter at the splanchnic‐nerve‐CC synapse (Feldberg et al. 1934). Cells were continuously perifused at 37°C; this was critical for three reasons: First, the Ca^2+^ transporters are highly sensitive to temperature; for instance in BCCs the plasmalemmal Na^+^‐dependent Ca^2+^ efflux works fivefold faster at physiological compared with room temperature (Villalobos et al. 2002) and the rate of the Na^+^‐dependent Ca^2+^ efflux through the mNCX is halved at room temperature with respect to 37°C (Montero et al. 2002); second, the rate of catecholamine release from BCCs is two‐ to threefold higher at 37°C, with respect to room temperature (Knight and Baker 1983; Kao and Westhead 1984; Bittner and Holz 1992; Walker et al. 1996; Gil et al. 2001; Haynes et al. 2007; Padin et al. 2013); and third, the rate of RRVP refilling after depolarizing pulses is accelerated at 37°C (Dinkelacker et al. 2000). ACh pulses were applied at 30 *μ*mol/L, the EC_50_ to elicit half‐maximal secretory responses from perifused BCCs (Cuchillo‐Ibanez et al. 2002). Another relevant issue was the repeated stimulation with 5‐sec pulses of ACh (12 pulses or more), given at 2‐min intervals to elicit docked pool depletion and its refilling or overfilling during the recovery intervals between ACh pulses.

**Figure 1.**
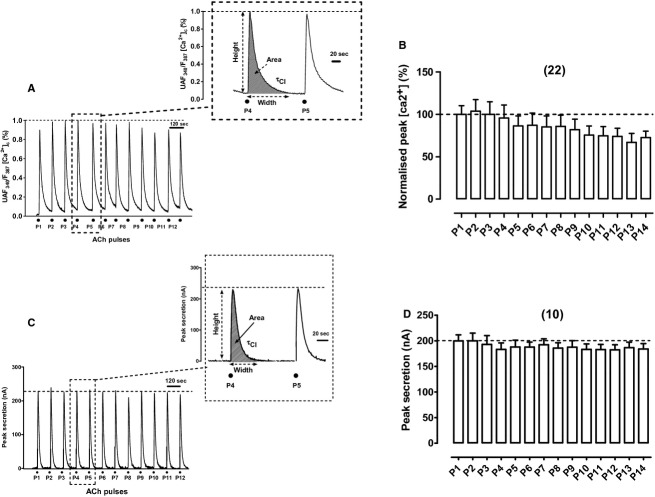
Cytosolic Ca^2+^ transients ([Ca^2+^]_c_) and catecholamine release transients elicited by repeated acetylcholine (ACh) pulses were quite reproducible. ACh pulses (30 *μ*mol/L ACh for 5 s, P1—P12) were sequentially applied at 2‐min intervals on single‐fura‐2‐loaded BCCs (to monitor [Ca^2+^]_c_ changes) perifused at 37°C, or on 5‐million BCC batches trapped in a microchamber (to monitor secretion changes), that were being continuously perifused with a Krebs‐Hepes solution at 37°C (see Methods for details). (A) Example record of the [Ca^2+^]_c_ transients elicited by ACh in a fura‐2‐loaded cell; inset shows the transient generated by the ACh pulses P4 and P5 at an expanded time scale. (B) Pooled averaged data on the normalized peak [Ca^2+^]_c_ height. (C) Example catecholamine release responses (in nA, ordinate) obtained from a BCC batch; inset shows the transients at an expanded time scale. (D) Averaged pooled results from the normalized secretion data obtained using the protocol of panel (C). Data in (B and D) are means ± SEM of the number of single cells or cell batches from three different cultures, shown in parentheses.

In an example fura‐2‐loaded‐BCC the repeated challenging with ACh pulses evoked quite reproducible [Ca^2+^]_c_ transients that were sustained during eight pulses (P1–P8); only at the final pulses (P9–P12) a mild decay in peak height was observed (Fig. 1A). Pulses given at 2‐min intervals permitted baseline recovery in practically all transients. When data from 22 cells were normalized and averaged, peak [Ca^2+^]_c_ height decayed by around 30% at pulses P10–P14 (Fig. 1B).

In an example of a perifused cell population, repeated exposure to ACh pulses evoked highly reproducible catecholamine secretion responses; with this stimulation pattern (2‐min intervals) the rate of secretion reached baseline in all pulses (P1–P12) (Fig. 1C). Averaged data from 10 cell batches produced quite stable secretory transients during the 30‐min period of intermittent cell challenging with ACh (Fig. 1D). This long‐term healthy status of perifused BCCs could be due to the physiological conditions used, as described above.

Reproducible bulk responses were further illustrated by monitoring their kinetic parameters, calculated as shown in the insets to Figure 1A and C, for the ACh pulses P4 and P5. Data of these parameters for control cells (peak height, width, area, and decay time constant, *τ*_cl_), are given in Table 1. All four parameters underwent less than 10% variation in P5 with respect to P4, suggesting that the selection of the P4–P5 interval to study drug action was correct.

**Table 1.** Kinetic parameters of the [Ca^2+^]_c_ and secretion transient responses elicited by ACh pulses given to perifused BCCs at 37°C, following the protocols shown in Figures 1–3.

Treatment	Height (peak) *		Width *		Area *		Clearance (*τ*) *	
	[Ca^2+^]_c_	Secretion	[Ca^2+^]_c_	Secretion	[Ca^2+^]_c_	Secretion	[Ca^2+^]_c_	Secretion
Control (P4)	95.6 ± 15.3	183 ± 12.5	97.8 ± 5.8	13.7 ± 0.8	91.6 ± 14.3	3.39 ± 0.16	19.2 ± 1.8	18.2 ± 1.2
**Control (P5)**	86.3 ± 11.9	188 ± 13.2	107 ± 6.4	13.3 ± 1.3	92.8 ± 16.1	3.31 ± 0.21	20.1 ± 1.9	18.3 ± 0.8
Change in P5 with respect to P4 (%)	**−9.7**	**+2.7**	**+9.4**	**−2.9**	**+1.3**	**−2.4**	**+4.7**	**+0.5**
Control (P4)	88.2 ± 9.4	175 ± 38	89 ± 9.7	12.5 ± 0.9	85.9 ± 10.8	2.77 ± 0.31	12.6 ± 4.4	15.7 ± 1.3
**Ru360 (P5)**	76.2 ± 8.8	465 ± 37*	136 ± 7.8*	11.6 ± 0.6	54.7 ± 19.7	7.1 ± 0.51	57.1 ± 10.3*	12.5 ± 0.8
Change in P5 with respect to P4 (%)	**−14**	**+166**	**+53**	**−7.2**	**−36**	**+256**	**+353**	**−20**
Control (P4)	91.3 ± 5.7	201 ± 11.5	106 ± 5.6	13 ± 0.6	97 ± 6	3.65 ± 0.12	19.7 ± 2.3	11.2 ± 0.5
**CGP37157 (P5)**	69.6 ± 5.2*	163 ± 9.7*	138 ± 4.7*	13.4 ± 0.7	86 ± 7.1	2.99 ± 0.09	38.6 ± 3.8*	11.6 ± 0.5
Change in P5 with respect to P4 (%)	**−24**	**−19**	**+30**	**+3**	**−11**	**−18**	**+96**	**+4**
Control (P4)	93.7 ± 1.2	193 ± 30.2	93.3 ± 2.3	13.1 ± 1.3	92.4 ± 3	3.3 ± 0.8	18.3 ± 1.6	15.7 ± 2.4
**Ru360 + CGP37157 (P5)**	71 ± 3.3	347 ± 47.8*	102.2 ± 4.7	14.7 ± 0.6	77.3 ± 7.6	6.3 ± 1.2	21.9 ± 3.2	15 ± 1.9
Change in P5 with respect to P4 (%)	**−24**	**+80**	**+10**	**+12**	**−16**	**+91**	**+20**	**−5**
Control (P4)	88.7 ± 1.4	188 ± 15.3	98.9 ± 5.2	13.1 ± 0.5	87 ± 3.7	3.2 ± 0.3	19.3 ± 2.4	16.5 ± 1.3
**FCCP (P5)**	78.3 ± 8.8	424 ± 25.3*	92.1 ± 6.8	13.6 ± 0.6	74.7 ± 12.3	7.2 ± 0.7*	9.16 ± 0.9*	16.1 ± 1.2
Change in P5 with respect to P4 (%)	**−12**	**+126**	**−7**	**+4**	**−14**	**+125**	**−53**	**−2**
Control (P4)	87 ± 9.4	181 ± 8.9	96.4 ± 3.1	13.2 ± 0.5	88.4 ± 9.3	2.96 ± 0.07	17.5 ± 1.2	14.6 ± 0.5
**Oligomycin plus rotenone (P5)**	48.9 ± 7.3	463 ± 20.6*	95.5 ± 6.6	13.6 ± 0.4	88.4 ± 14.3	8.34 ± 0.30*	18.6 ± 2.2	13.4 ± 0.4
Change in P5 with respect to P4 (%)	**−44**	**+156**	**−1**	**−1**	**0**	**+182**	**+6**	**−8**

The parameters were calculated as indicated in the insets of Figure 1A and C, and correspond to pulses P4 (control, before giving the compound) and P5 (applied to cells that were perifused with the compounds for 2 min before and during the ACh pulse); drug treatment and % of variation were emphasized in bold.

**Figure 2.**
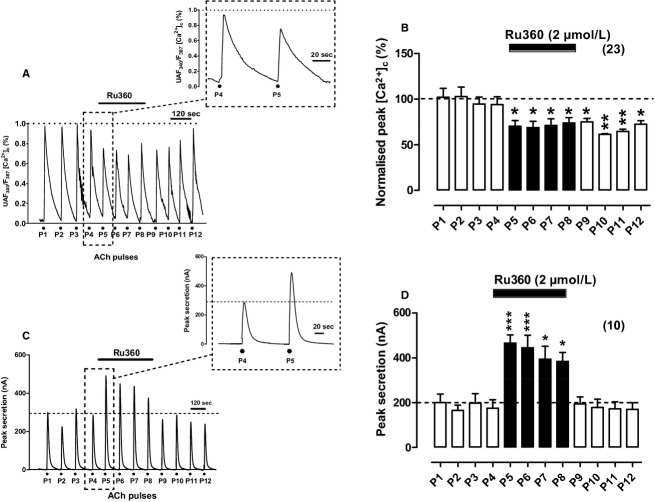
Effects of Ru360 on [Ca^2+^]_c_ and secretion responses elicited by repeated cell challenging with acetylcholine (ACh). Experimental conditions and ACh challenging are as in Figure 1, except for the fact here Ru360 was perifused with the Krebs solution since 1 min before P5 and was withdrawn 1 min after P8 (top horizontal bars). (A) Example record of the [Ca^2+^]_c_ transients elicited by ACh in a fura‐2‐loaded BCC; inset shows the transients generated by the ACh pulses P4 and P5 at an expanded time scale. (B) Pooled averaged data on the normalized peak [Ca^2+^]_c_ amplitude. (C) Example catecholamine release responses (in nA, ordinate) obtained from a BCC batch; inset shows the transients elicited by ACh pulses P4 and P5 at an expanded time scale. (D) Averaged pooled results from the secretion data obtained using the protocols of panel (C). Data in (B and D) are means ± SEM of the number of cells and cell batches from three different cultures, shown in parentheses. *P <**0.05, ***P <**0.001 with respect to P4.

**Figure 3.**
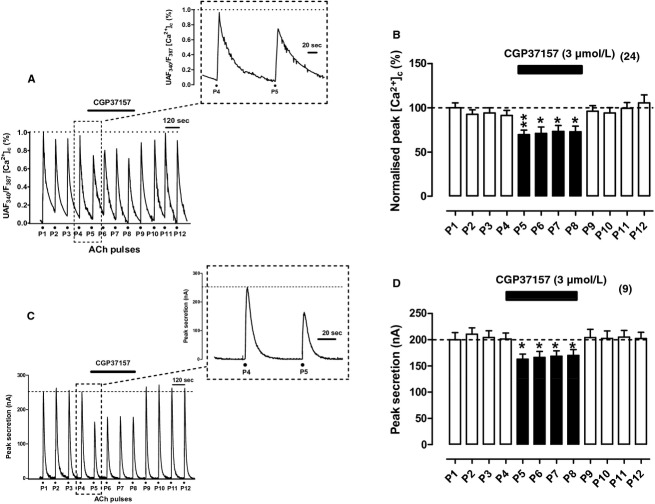
Effects of CGP37157 on [Ca^2+^]_c_ and secretion transients elicited by repeated cell challenging with ACh. Experimental conditions and stimulation with ACh were as in Figure 1, except for the fact here CGP37157 (3 *μ*mol/L) was cell perifused since 1 min before P4 to 1 min after P8 (top horizontal bars). (A) Example record of the [Ca^2+^]_c_ transients elicited by ACh in a fura‐2‐loaded BCC; inset shows the transients generated by the ACh pulses P4 and P5 at an expanded time scale. (B) Pooled averaged data on the normalized peak [Ca^2+^]_c_ amplitude, calculated from experiments as that shown in (A). (C) Example catecholamine release responses (in nA, ordinate) obtained from a BCC batch; inset shows the transients elicited by ACh pulses P4 and P5 at an expanded time scale. (D) averaged data from the secretion responses obtained using the protocols of panel (C). Data in (B and D) are means ± SEM of the number of single cells ([Ca^2+^]_c_) and cell batches (secretion) from three different cultures, shown in parentheses. *P <**0.05, **P <**0.01 with respect to P4.

**Figure 4.**
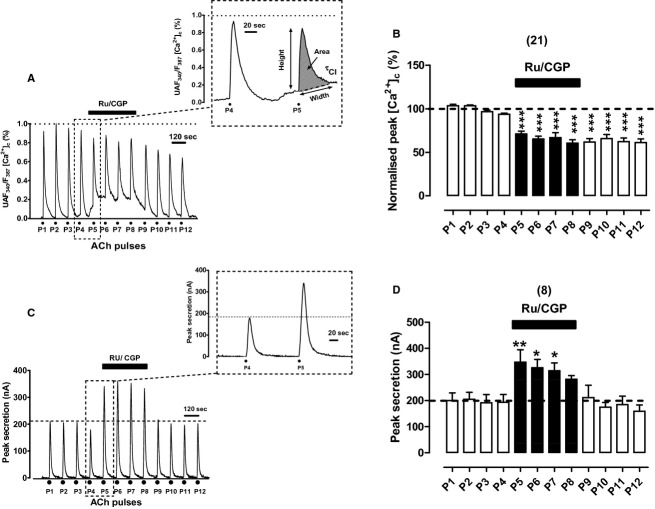
Effects of Ru360 (2 *μ*mol/L) and CGP37157 (3 *μ*mol/L) given in combination (Ru/CGP) on [Ca^2+^]_c_ and secretion transients elicited by repeated cell challenging with ACh. Experimental conditions and stimulation with ACh were as in Figure 1, except for the fact here Ru/CGP was cell‐perifused since 1 min before P5 to 1 min after P8 (top horizontal bars in each panel). (A) Example record of the [Ca^2+^]_c_ transients elicited by ACh in a fura‐2‐loaded BCC; inset shows the transients generated by the ACh pulses P4 and P5 at an expanded time scale. (B) Pooled averaged data on the normalized peak [Ca^2+^]_c_ amplitude. (C) Example catecholamine release responses (in nA, ordinate) obtained from a BCC batch; inset shows the transients elicited by ACh pulses P4 and P5 at an expanded time scale. (D) Averaged data from the secretion responses obtained using the protocols of panel (C). Data in (B and D) are means ± SEM of the number of single cells ([Ca^2+^]_c_) and cell batches (secretion) from three different cultures, shown in parentheses. *P <**0.05, **P <**0.01 with respect to P4.

**Figure 5.**
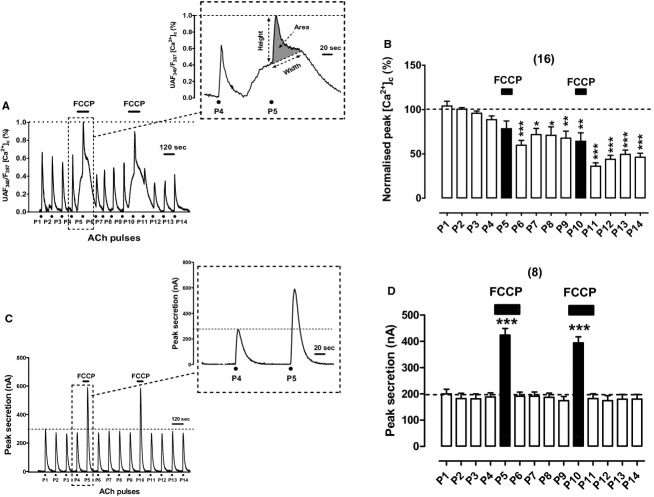
Effects of FCCP (2 *μ*mol/L) on [Ca^2+^]_c_ and secretion transients elicited by repeated cell challenging with ACh. Experimental conditions and stimulation with ACh are as in Figure 1, except for the fact here FCCP was cell‐perifused since 1 min before, and during P5 (top horizontal bars in each panel). (A) example record of the [Ca^2+^]_c_ transients elicited by ACh in a fura‐2‐loaded BCC; inset shows the transients generated by the ACh pulses P4 and P5 at an expanded time scale. (B) Pooled averaged data on the normalized peak [Ca^2+^]_c_ amplitude. (C) Example catecholamine release responses (in nA, ordinate) obtained from a BCC batch; inset shows the transients elicited by ACh pulses P4 and P5 at an expanded time scale. (D) Averaged data from the secretion responses obtained using the protocols of panel (C). Data in (B and D) are means ± SEM of the number of single cells ([Ca^2+^]_c_) and cell batches (secretion) from three different cultures, shown in parentheses. ***P <**0.001 with respect to P4 and P9.

**Figure 6.**
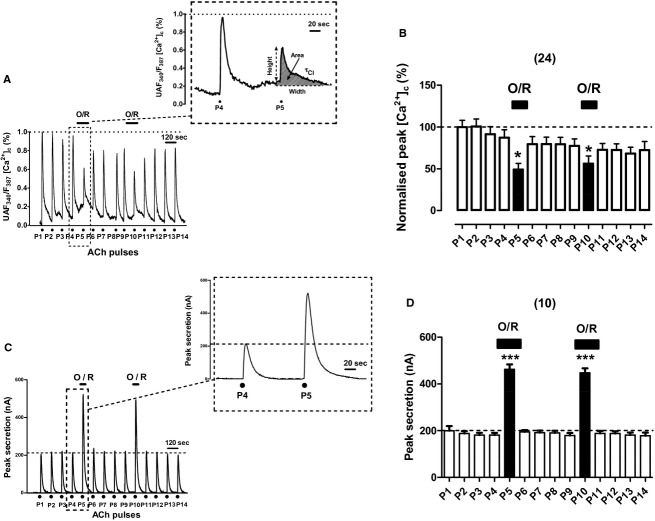
Effects of oligomycin (4 *μ*mol/L) and rotenone (3 *μ*mol/L) given in combination (O/R) on [Ca^2+^]_c_ and secretion transients elicited by repeated cell challenging with ACh. Experimental conditions and stimulation with ACh are as in Figure 1, except for the fact here O/R was cell‐perifused since 1 min before and during P5 or P10 (top horizontal bars in each panel). (A) Example record of the [Ca^2+^]_c_ transients elicited by ACh in a fura‐2‐loaded BCC; inset shows the transients generated by the ACh pulses P4 and P5 at an expanded time scale. (B) Pooled averaged data are on the normalized peak [Ca^2+^]_c_ amplitude. C, example catecholamine release responses (in nA, ordinate) obtained from a BCC batch; inset shows the transients elicited by ACh pulses P4 at an expanded time scale. (D) Averaged data from the secretion responses obtained using the protocols of panel (C). Data in (B and D) are means ± SEM of the number of single cells ([Ca^2+^]_c_) and cell batches (secretion) from three different cultures, shown in parentheses. ***P <**0.001 with respect to P4 and P9.

* Height, response amplitude to peak: in the case of [Ca^2+^]_c_ it was normalized to 100% (Fura‐2 ratios) and in the case of secretion is given as nA current generated by the oxidation of the released catecholamine.

* Width of the response transient signal is given in seconds.

* Area represents the integrated [Ca^2+^]_c_ or secretion transients (bulk CRRs) expressed as time‐fold the fura‐2‐fluorescence ratios or as *μ*C (nA × s), respectively.

* *τ* for the clearance (*τ*_cl_) is the time constant for the decay of the [Ca^2+^]_c_ and secretion responses given in s^−1^. Data are means ± SEM (calculated from the experiments of Figs. 1–6). Statistical analyses were carried out with ANOVA one‐way test, and Dunnett′s post hoc test; *P < 0.05 with respect to P1.

### Effects of Ru360 on [Ca^2+^]_c_ and secretion transients elicited by repeated cell challenging with ACh

To perform these experiments we used a concentration of 2 *μ*mol/L Ru360; this was double than the concentration of 1 *μ*mol/L usually used to target the mCUP in permeabilized cells (Fluegge et al. 2012; Mallilankaraman et al. 2012). In intact BCCs, 2 *μ*mol/L Ru360 perifused for 2–8 min was capable of producing clear‐cut effects on [Ca^2+^]_c_ and secretion transients indicating that the compound entered the cells.

If subplasmalemmal mitochondria take up Ca^2+^ with high efficiency upon ACh challenging of BCCs (Montero et al. 2000), it follows that blockade of the uniporter with Ru360 should disturb the [Ca^2+^]_c_ transient kinetics. In the example cell of Figure 2A, the compound produced a decrease in peak height and a slower decay rate (see the transients at inset, with and extended time scale). This is better illustrated in the bar diagram of Figure 2B, that was graphed with pooled data from 23 cells; the decay was more pronounced than the equivalent decay of P5–P8 pulses of control cells (compare Figs. 1B, 2B), and the [Ca^2+^]_c_ transient did not recover upon removal of Ru360 (P9–P12 ACh pulses). Table 1 indicates that in the presence of Ru360 (ACh pulse P5), the averaged peak height was reduced by 14%, the width was enhanced by 55%, the area was reduced by 35%, and the *τ*_cl_ was augmented by 340%, indicating that Ru360 considerably delayed the clearance of the [Ca^2+^]_c_ transient elicited by ACh.

Secretion responses were drastically augmented in the presence of Ru360, as shown in the original record from an example cell batch (Fig. 2C) and in the pooled data from 10 cell batches (Fig. 2D). Although this enhanced response was maintained during the four pulses (P5–P8) in which Ru360 was present, it, however, tended to decline in successive pulses. The kinetic parameters of the response of P5 (in the presence of Ru360) with respect to P4 (in its absence) were as follows (Table 1): 170% increase in the peak height, 130% increase in area and 30% decrease in *τ*_cl_; because the width did not change, the enhanced response seemed to be essentially due to an augmented rate of initial secretion.

### Effects of CGP37157 on [Ca^2+^]_c_ and secretion transients elicited by repeated cell challenging with ACh

CGP37157 is widely being used to inhibit the mNCX at concentrations of 10 *μ*mol/L in neuronal cultures (Medvedeva et al. 2008; Ryan et al. 2009) and at 3–15 *μ*mol/L in brain slices (Kovacs et al. 2005; Nicolau et al. 2010). In permeabilized HeLa cells, CGP37157 blocks the mitochondrial Ca^2+^ efflux through the mNCX with an IC_50_ of 1.6 *μ*mol/L (Hernandez‐SanMiguel et al. 2006). The selectivity of CGP37157 is limited and it has been shown to target also the VACCs. For instance, in dorsal root ganglion neurons CGP37157 blocks the K^+^‐elicited [Ca^2+^]_c_ transients with an IC_50_ of 4 *μ*mol/L (Baron and Thayer 1997). On the other hand, 1 *μ*mol/L CGP37157 does not affect *I*_Ca_ in isolated rat ventricular myocytes while it causes mNCX inhibition in isolated heart mitochondria with an IC_50_ of 0.36 *μ*mol/L (Cox et al. 1993). However, in rat atrial myocytes the compound inhibits *I*_Ca_ with an IC_50_ of 0.27 *μ*mol/L (le Thu et al. 2006). In rat CCs, 10 *μ*mol/L CGP37157 slows the Ca^2+^ loss from mitochondria (Babcock et al. 1997). Finally, in BCCs, the cell model used in this study, CGP37157 at 10 *μ*mol/L causes 18% inhibition of *I*_Ca_, and 60% blockade at 30 *μ*mol/L; at 3 *μ*mol/L, I_Na_ blockade was less than 10% and at 5 *μ*mol/L CGP37157 did not affect the veratridine‐elicited [Ca^2+^]_c_ oscillations (Nicolau et al. 2009). In this study we choose 3 *μ*mol/L CGP37157 to test its effects on [Ca^2+^]_c_ and secretion transients elicited by ACh; according to the literature commented above, it was expected that this concentration would target quite selectively the mNCX of BCCs.

In BCCs stimulated with high K^+^, the release into the cytosol of the Ca^2+^ taken up by mitochondria at 37°C is considerably slowed down by CGP37157 (Montero et al. 2000). Therefore, it was expected that this mNCX blocker could affect both the [Ca^2+^]_c_ and secretion responses to ACh. This possibility was explored in experiments with protocols similar to those used previously for Ru360. For instance, in the example fura‐2‐loaded cell of Figure 3A, CGP37157 at 3 *μ*mol/L caused a prompt decrease in the peak height and slowed down the decay of the [Ca^2+^]_c_ transient; these changes were better seen when responses to ACh pulses P4 and P5 were graphed at an expanded time scale (see inset of Fig. 3A). Pooled results from 24 cells showed a 30% decrease in peak [Ca^2+^]_c_ amplitude; such decrease was sustained along the four ACh pulses in which CGP37157 was present and the response recovered its initial height upon washout of the compound (Fig. 3B). Peak height decrease was accompanied by 40% increase in the width, and 50% increase in *τ*_cl_; the area was not significantly altered (Table 1).

The secretion transients were also decreased in the presence of CGP37157 (see example cell batch of Fig. 3C); full response recovery was achieved upon compound washout. These changes were fairly reproduced in the nine cell batches averaged in Figure 3D. The height and area of secretion responses were decreased by 20%, and the width and *τ*_cl_ showed no changes. Thus, it seemed that CGP37157 only produced a mild reduction in the total amount of released catecholamine without altering the rate and duration of the secretory responses to ACh.

### Effects of combined Ru360 and CGP37157 on [Ca^2+^]_c_ and secretion transients elicited by repeated cell challenging with ACh

It was expected that the simultaneous blockade of Ca^2+^ uptake into, and its subsequent release from mitochondria could elicit greater alterations of the [Ca^2+^]_c_ and secretion responses to ACh. Hence, we decided to use Ru360 at 2 *μ*mol/L and CGP37157 at 3 *μ*mol/L in combination (Ru/CGP). The first interesting observation was that cell exposure to Ru/CGP immediately enhanced the basal [Ca^2+^]_c_, as shown in the original trace of the example cell of Figure 4A. Superimposed on this elevated basal [Ca^2+^]_c_ is the [Ca^2+^]_c_ transient elicited by ACh P5, that if measured with respect to the new baseline was 40% smaller than P4. The second interesting finding was that basal [Ca^2+^]_c_ remained elevated along the time period Ru/CGP was present (8 min); at ACh pulses P6, P7, and P8, the transients had smaller amplitudes than controls P1–P4. Upon Ru/CGP washout, baseline [Ca^2+^]_c_ recovered its initial level; in spite of this, the transient remained around 40% lower than the initial responses. The inset in Figure 4A shows the control P4 trace as well as the P5 trace superimposed on the elevated basal [Ca^2+^]_c_ in the presence of Ru/CGP. Thus, in resting conditions it seems that the mCC is effectively working to maintain low the basal [Ca^2+^]_c_. The simple blockade of one of the two Ca^2+^ transporters is insufficient to elevate basal [Ca^2+^]_c_; blockade of both transporters is therefore needed to suppress the mCC and to enhance basal [Ca^2+^]_c_. This is surprising given the reported low Ca^2+^ affinity of the mCUP in BCCs (Uceda et al. 1995; Montero et al. 2000).

Concerning secretion it is worth mentioning that combined Ru/CGP caused a pronounced increase in peak height; however, it was curious that the initial level of baseline secretion was recovered after each ACh pulse (see in Fig. 4C the ACh pulses P5 to P8 given in the presence of Ru/CGP). Therefore, the enhanced basal [Ca^2+^]_c_ was not paralleled by an augmented rate of basal secretion. The pronounced augmentation of height and area of the secretion response in P5 with respect to P4, is better seen in the inset to Figure 4C. Pooled results from eight BCC batches corroborate the drastic increase in the ACh secretory response; however, such increase tended to decline in the successive ACh pulses (P5–P8) given in the presence of Ru/CGP (Fig. 4D). The kinetic parameters of P5 responses (in the presence of Ru/CGP) with respect to P4 responses (in the absence of the compounds) were as follows (Table 1): 80% increase in peak height, and 200% increase in peak area with minor changes in the width and *τ*_cl_. This indicates that the combined blockade of mitochondrial Ca^2+^ influx and efflux during ACh challenging produces augmented secretion of total catecholamine without altering the kinetics of responses, indicating that more vesicles were available for secretion.

### Effects of FCCP on [Ca^2+^]_c_ and secretion transients elicited by repeated cell challenging with ACh

Protonophores FCCP and CCCP enhance exocytosis elicited by depolarizing pulses in voltage‐clamped BCCs (Giovannucci et al. 1999) as well as in BCC batches stimulated with ACh or high K^+^ (Montero et al. 2000; Cuchillo‐Ibanez et al. 2004). However, the correlation between the [Ca^2+^]_c_ and secretion responses triggered by ACh at 37°C has not yet been explored in BCCs. Figure 5A shows an example record of the ACh‐elicited [Ca^2+^]_c_ responses in an example cell. Of note was the pronounced, slow‐developing elevation of the basal [Ca^2+^]_c_ upon cell exposure to 2 *μ*mol/L FCCP during the 1‐min period preceding P5. Superimposed on this elevated basal [Ca^2+^]_c_ was a smaller but faster [Ca^2+^]_c_ response elicited by ACh. Both the slow elevated basal [Ca^2+^]_c_ and the ACh [Ca^2+^]_c_ transient response are better seen at the inset of Figure 5A, where the responses to P4 and P5 pulses are represented at an expanded time scale. Pooled results from 16 cells showed that the normalized peak [Ca^2+^]_c_ tended to decay with time and that in the presence of FCCP the net peak [Ca^2+^]_c_ generated by ACh at P5 was similar to P4. Additionally, transient duration and area were also similar; only the clearance was faster (about twofold) in P5 with respect to P4 (Table 1).

Worth of note was the fact FCCP did not augment the rate of basal catecholamine release during the 1 min preceding the ACh pulses P5 and P10, as illustrated in the example cell batch record of Figure 5C; this is better seen at the inset showing P4 and P5 responses with at expanded time scale. Pooled results from eight cell batches illustrate the large potentiation of secretion responses in cells exposed to FCCP and challenged with ACh at P5 and P10 (Fig. 5D). The averaged kinetic values for secretion at P5 with respect to P4 were as follows: 2.25‐fold increase in peak height and peak area and no change in width and *τ*_cl_ (Table 1). It seems therefore that enhanced responses in the presence of FCCP are due to a higher total catecholamine release, essentially represented by a higher peak height.

### Effects of combined oligomycin plus rotenone on [Ca^2+^]_c_ and secretion transients elicited by repeated cell challenging with ACh

Protonophores might disturb not only the Ca^2+^ handling by mitochondria (Herrington et al. 1996) but also the Ca^2+^ stored in other organelles such as the endoplasmic reticulum (Goeger and Riley 1989; Seidler et al. 1989; Lim et al. 2006) or chromaffin vesicles (Haynes et al. 2006). Therefore, combined oligomycin at 4 *μ*mol/L to block mitochondrial ATP synthase and rotenone at 4 *μ*mol/L to block complex I of the respiratory chain (O/R) were used to abort mCC. Figure 6A shows the original [Ca^2+^]_c_ transients elicited by repeated ACh pulsing of an example fura‐2‐loaded BCC. As in the case of Ru/CGP and FCCP, O/R also caused an elevation of basal [Ca^2+^]_c_; however, such elevation was milder than that elicited by FCCP, as shown in the inset of Figure 6A. Furthermore, the ACh [Ca^2+^]_c_ transient was slightly reduced in the presence of O/R, as shown in pulses P5 and P10 in Figure 6B, showing bar graph with pooled data from 24 cells. The kinetic parameters of P5 (in the presence of O/R) were similar to those of P4; only a 44% decrease in peak height was noticed (Table 1).

In spite of baseline elevation of [Ca^2+^]_c_, combined O/R did not affect baseline secretion (Fig. 6C). However, O/R caused a drastic increase in secretion at pulses P5 and P10 of the example cell batch of Figure 6C; this increase is better seen at the inset where responses P4 (control) and P5 (in the presence of O/R) are displayed. Pooled data from nine cells batches are graphed in Figure 6D; the potentiation of secretion by O/R was produced at pulses P5 and P10 within the same batch of cells. Peak height was enhanced by 250% and the secretion transient area was augmented by 270% in the presence of O/R; no significant changes were observed in the width and *τ*_cl_ of the response (Table 1).

### Effects of combined Ru360 and CGP37157 on near‐membrane calcium transients elicited by ACh

The Ca^2+^ probe fura‐2 measure the bulk [Ca^2+^]_c_ changes in the low micromolar range. However, during depolarization of CCs, HCMDs of 10 *μ*mol/L or more occur at subplasmalemmal areas, near exocytotic sites (Neher 1998; Garcia et al. 2006; Garcia‐Sancho et al. 2012). We therefore decided to monitor the near‐membrane changes of Ca^2+^ ([Ca^2+^]_nm_) with the low‐Ca^2+^ affinity probe FFP18 (Etter et al. 1996).

**Figure 7.**
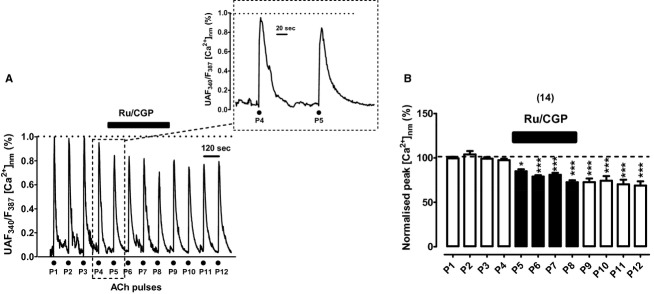
Effects of combined Ru360 (2 *μ*mol/L) and CGP37157 (3 *μ*mol/L) on the near‐membrane cytosolic Ca^2+^ transients ([Ca^2+^]_nm_), elicited by repeated pulses of acetylcholine (ACh, 30 *μ*mol/L, 5‐sec pulses) applied to bovine chromaffin cells (BCCs) that have been perifused with a Krebs‐Hepes solution containing 2 mmol/L Ca^2+^ at 37°C, in a continued manner. Cells were loaded with 10 *μ*mol/L FFP18 before the experiments (see Methods). (A) Example record of the [Ca^2+^]_nm_ transients (ordinate, normalized to the maximal response) elicited by ACh (dots at the bottom) before (P1–P4), during Ru/CGP perifusion (P5–P8), and after washout (P9–P12); inset shows the transients generated by the ACh pulses P4 (in the absence) and P4 (in the presence of Ru/CGP applied 2 min before) at an expanded scale. (B) Pooled averaged data on the normalized peak [Ca^2+^]_nm_ amplitude; they are means ± SEM of the number of cells shown in parentheses. *P <**0.05, ***P <**0.001 with respect to P4.

Upon challenging with repeated ACh pulses, quite reproducible transients of [Ca^2+^]_nm_ were produced, as illustrated in the records taken from an example cell loaded with FFP18 (pulses P1–P4 of Fig. 7A). All transients reached baseline before the next ACh pulse was applied. The cell perifusion with Ru/CGP produced a mild gradual decay of peak height (pulses P5–P8 of Fig. 7A); after compound washout the [Ca^2+^]_nm_ did not recover its initial amplitude. This effect was qualitatively similar to that produced by Ru/CGP in the fura‐2‐loaded cell of Figure 4A. However, there was a sharp difference between the [Ca^2+^] monitored by the two probes. With fura‐2, a sustained mild elevation of basal [Ca^2+^]_c_ was produced (Fig. 1A and inset); however, with FFP18 the basal [Ca^2+^]_nm_ remained unaltered along the entire experiment (Fig. 7A and inset). Pooled results from 14 cells are summarized in Figure 7B; a significant 20–30% decrease in peak [Ca^2+^]_nm_ was produced by Ru/CGP. This could be due to inactivation of VACCs that may occur during mCC blockade (Hernandez‐Guijo et al. 2001).

### Effects of Ru360 and CGP37157 on the membrane potential

We performed these experiments to test the possibility that Ru360 and CGP37157 applied separately or jointly, could affect the membrane potential (Em); alteration of cell excitability by the compounds could contribute to the effects on [Ca^2+^]_c_ and secretion transients elicited by ACh.

**Figure 8.**
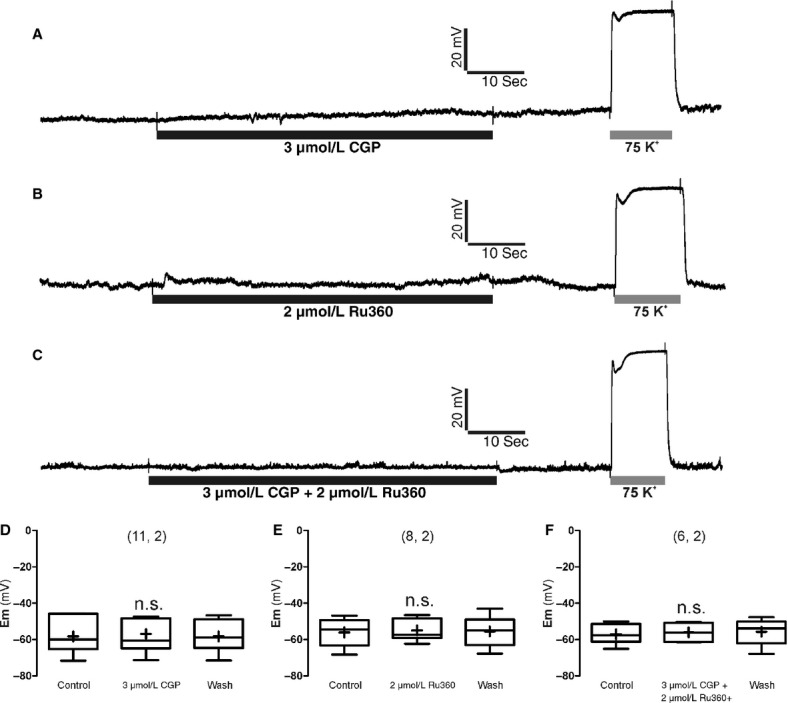
Effects of combined Ru360 (2 *μ*mol/L) and CGP37157 (3 *μ*mol/L) on the resting membrane potential (Em) monitored in current‐clamped BBC. (A–C) are records taken from three BCCs exposed to the compounds at the concentrations and time periods indicated at the bottom horizontal bars below each trace. K^+^ (75 mmol/L, low Na^+^) was given at the end of each experiment as a positive control of cell depolarization. (D–F) are the summary of pooled data from the number of cells and cultures shown in parentheses on top of each set of box‐and‐whiskers plots (the line inside the box depicts median values, the cross inside the box represent mean values, the size of the box is given by the distance between the 25th and the 75th percentiles; upper “whisker” reach the 90th percentile and lower “whisker” the percentile 10th). Em, resting membrane potential before, during, and after washout of the compounds. n.s., nonsignificant statistical difference compared with control.

Current‐clamped BCCs had a resting Em of around −60 mV, and they seldom fired an isolated action potential; these data are consistent with those from previous reports (Orozco et al. 2006; de Diego 2010). Figure 8 shows original Em recordings taken from three cells exposed to the compounds at the concentrations used to explore their effects on [Ca^2+^]_c_ and secretion transients triggered by ACh. Given separately or in combination, CGP37157 and Ru360 did not alter the resting Em (panels A–C). However, cell perifusion of the cells with a saline solution containing 75 mmol/L K^+^ caused the expected immediate depolarization to 0 mV of BCCs. Mean pooled data from 6 to 11 cells corroborated the lack of effects of Ru360 and CGP37157, either given alone or in combination, on the resting Em of BCCs.

### Effects of combined Ru360 and CGP37157 on the size of the readily releasable vesicle pool

Once loaded with their release cargo, chromaffin vesicles approach the plasma membrane until they make contact with it; they are then said to be docked. There they undergo several priming steps until they get ready for exocytosis forming the readily releasable pool (Neher 1998; Sorensen 2004). To measure the effect of Ru/CGP on the size of this pool we utilized perforated‐patch clamp and the double‐pulse protocol that consists in applying two shorts depolarizing pulses a few milliseconds apart, and measuring the cell membrane capacitance changes (ΔCm) thus produced as an estimate of exocytosis. The rationale behind this experiment is that a sufficiently strong first stimulus will cause the fusion of most of all the vesicles ready to release (ΔCm1); hence, if a second stimulus is applied before the pool is replenished, the second secretory response (ΔCm2) will show depression (Gillis et al. 1996). We applied depolarizing pulses adjusting the potential so that *I*_Ca_ was similar for both pulses that were separated 100 msec and had the duration of 100 msec.

**Figure 9.**
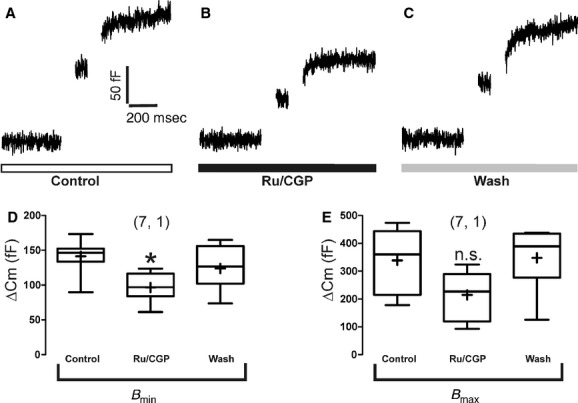
Effects of combined Ru360 (2 *μ*mol/L) and CGP37157 (3 *μ*mol/L) on the ready‐release vesicle pool (RRVP) sizes estimated with the double‐pulse protocol in voltage‐clamped BBCs. Panel (A) shows original Cm control recordings of the 100 msec depolarization double‐pulse protocol. Panel (B) shows original Cm recordings applied to cells, which were perifused with the mixture of compounds for 4 min before and during the double‐pulse protocol. Panel (C) shows original Cm recordings after washout of compounds of the 100 msec depolarization double‐pulse protocol. Pooled results of 7 BCCs from one cell culture, used to compute the effect of Ru/CGP in the RRVP are graphed in panels (D) (*B*_min_) and (E) (*B*_max_). ANOVA one‐way test, and Tukey post hoc analyses; *P <**0.05 with respect to saline control.

Figures 9A–C show the ΔCm responses of a voltage‐clamped BCC stimulated three times with the double‐pulse protocol, before, during perifusion of Ru/CGP and after washout of these compounds. The size of the RRVP before compound application oscillated between 142 ± 10 fF (*B*_min_) and 339 ± 43 fF (*B*_max_). *B*_min_ decreased by 31% in the presence of Ru/CGP; however, *B*_max_ was not significantly altered (Fig. 9D and E). Of note is the observation that Ru/CGP did not affect *I*_Ca_.

## Discussion

Calcium handling by mitochondria was first suggested to shape the [Ca^2+^]_c_ signals generated by depolarizing pulses of millisecond to few seconds duration in voltage‐clamped rat CCs (Herrington et al. 1996). Later on it was shown that suppression of mCC with protonophores augmented exocytosis monitored as an increase in membrane capacitance in voltage‐clamped BCCs; this suggested that mitochondria were also regulating exocytosis (Giovannucci et al. 1999). The secretory response evoked by K^+^ pulses of few seconds duration is also augmented by protonophores in BCCs (Montero et al. 2000; Villalobos et al. 2002; Cuchillo‐Ibanez et al. 2004) as well as in rat CCs (Miranda‐Ferreira et al. 2009). Thus, there is quite solid evidence that Ca^2+^ buffering by mitochondria plays a relevant role in shaping the [Ca^2+^]_c_ and exocytotic transients generated by depolarization of CCs. However, the question remains on whether and how mitochondria could contribute to the regulation of only the last steps of exocytosis requiring as high as 50 *μ*mol/L [Ca^2+^]_c_ underneath the plasma membrane (Neher 1998), or if they may also contribute to the shaping of the much lower [Ca^2+^]_c_ elevations that are required for vesicle transport and the refilling of vesicles pools docked at the plasmalemma (von Ruden and Neher 1993).

Measuring the changes of matrix mitochondrial Ca^2+^ ([Ca^2+^]_m_) with aequorin with different affinities for Ca^2+^, we suggested the existence of two different mitochondrial pools, M1 and M2, which take up Ca^2+^ at very different rates in response to activation of VACCs of BCCs. Pool M1 takes up Ca^2+^ at a rate of about 50 *μ*mol/L/s, 30% of the mCUP Vmax, whereas uptake by pool M2 is about 150 times slower (Montero et al. 2000; Villalobos et al. 2002). According to the saturation kinetic parameters estimated in permeabilized BCCs, the rates of mitochondrial Ca^2+^ uptake correspond to about 20 and 3 *μ*mol/L for pools M1 and M2, respectively. We believe that M1 corresponds to mitochondria close the plasma membrane, which sense high‐Ca^2+^ microdomains formed close to the mouth of VACCs and takes up most of the very large amounts of Ca^2+^ entering through those channels, thus muffling the progression of the Ca^2+^ wave toward the cell core. On the other hand, pool M2 would sense much smaller [Ca^2+^]_c_ at the cell core and will take up a minor fraction of the load (Garcia‐Sancho et al. 2012). Electron microscopy x‐ray microanalysis of frog sympathetic neurons, after stimulation with high K^+^, also revealed the existence of two mitochondria pools with different Ca^2+^ contents (Pivovarova et al. 1999) and so did aequorin in mouse sympathetic neurons (Nunez et al. 2007) and pancreatic beta cells (Quesada et al. 2008). Furthermore, in pancreatic acinar cells, entry of Ca^2+^ through plasma membrane also causes a preferential Ca^2+^ uptake in subplasmalemmal mitochondria (Park et al. 2001).

Repeated pulsing with ACh at 37°C produced reproducible catecholamine release responses for a long time period (Fig. 1). This indicates the existence of two simultaneous processes: on the one hand, ACh caused the release of catecholamine from mature vesicles docked at the plasmalemma; on the other, during ACh pulse intervals (2 min) vesicle transport from the cell core to subplasmalemmal sites must be occurring to refill the docked vesicle pools that had been exhausted by the previous ACh pulse, thus securing that the next ACh pulse trigger a similarly healthy secretory response. Ru360 augmented the ACh secretory response (Fig. 2) likely by two mechanisms: first, by blocking Ca^2+^ uptake into the M1 pool; and second, by delaying the clearance of the ACh load. The first mechanism implied a higher [Ca^2+^]_c_ microdomain; this could not be demonstrated with fura‐2 because this probe is measuring the bulk cytosolic [Ca^2+^]_c_. Neither the near‐membrane Ca^2+^ probe could sense a higher ACh [Ca^2+^]_nm_ transient in the presence of Ru/CGP; rather the peak height was mildly decreased. We interpret this finding as Ca^2+^‐dependent inactivation of VACCs that is accelerated upon blockade of the mCC (Hernandez‐Guijo et al. 2001). In spite of this, the ACh secretory response was enhanced 2.5‐fold in the presence of Ru360, an effect that may obviously be associated to the 3.5‐fold delay in the clearance of the ACh [Ca^2+^]_c_ transient (Table 1). It is plausible that such delay is due to Ca^2+^ redistribution by the mitochondrial M2 pool that may therefore be stimulating vesicle transport toward exocytosis sites. It should be noted that the secretory response in our perifusion system (five million BCCs trapped in the microchamber) takes about a minute after the 5‐sec ACh pulse to recover baseline levels. During this period, which overlays the ACh pulse, the long delay of the [Ca^2+^]_c_ clearance could explain the higher total secretion.

That CGP37157 diminished mildly the ACh‐elicited [Ca^2+^]_c_ and secretion transients was an expected outcome; inhibition of the mNCX by the compound would mitigate the rate of release of mitochondrial Ca^2+^ into the cytosol, leading to lower rate of vesicle transport and decreased secretion. Combined Ru/CGP, however, decreased the ACh [Ca^2+^]_c_ transient but the potentiation of secretion remained. Again we can implicate the Ca^2+^‐dependent inactivation of VACCs to explain this apparent paradox.

If applied separately, neither Ru360 nor CGP37157 affected basal [Ca^2+^]_c_. However, if given together the basal [Ca^2+^]_c_ underwent a mild elevation that was sustained along the 6‐min time period of cell perifusion with Ru/CGP (Fig. 4). This indicates that the mCC must be completely blocked (i.e., endless Ca^2+^ uptake through the mCUP and its subsequent release through the mNCX) in order to release the Ca^2+^ stored in the mitochondrial matrix. In support of this were the experiments with FCCP and O/R that also blocks the mCC and hence, caused an elevated basal [Ca^2+^]_c_. The fact Ru/CGP did not elevate [Ca^2+^]_nm_ suggests that the elevation of the bulk basal [Ca^2+^]_c_ is unrelated to mitochondrial pool M1; rather, it suggests that the basal [Ca^2+^]_c_ elevation is associated with the mCC of the M2 mitochondrial pool. It seems, therefore, that this low‐rate of Ca^2+^ cycling across the M2 pool is contributing to Ca^2+^ redistribution to the cell core, to activate vesicle transport toward exocytotic sites. Thus, the drastic potentiation of ACh secretory responses by Ru/CGP (Fig. 4), FCCP (Fig. 5) and O/R (Fig. 6) may have a double component: first, inhibition of Ca^2+^ buffering of M1 to augment exocytosis of docked vesicles; and second, blockade of M2 mCC to augment the trafficking of new vesicles toward exocytotic sites.

One last point deserves attention. The double‐pulse experiments with measurement of ΔCm suggests that Ru/CGP did not augment the readily releasable pool (Neher 1998; Sorensen 2004). These experiments are measuring exocytosis elicited by 100‐ms depolarizing pulses, a 50‐fold lower time range that the ACh pulses applied to cell populations. Assuming a mean capacitance of ∼2 fF per dense‐core vesicle in BCCs (estimates range from 1.3 to 2.7 fF) (Neher and Marty 1982; Chow et al. 1996), the size of the RRVP oscillated between 71 vesicles (*B*_min_) and 170 vesicles (*B*_max_). The maximum number of vesicles of the RRVP, in cells exposed to Ru/CGP was similar, indicating that the pool was unchanged. It seems therefore that enhanced secretion elicited by the blockade of the mCC is due to the continued supply of new vesicles to a stable docked pool that is undergoing exocytosis during repeated ACh stimulation at 37°C, a kind of facilitation of exocytosis occurring during mild elevations of [Ca^2+^]_c_ before stimulus application.

Mitochondria are a part of a functional triad that also includes VACCs and the endoplasmic reticulum (ER); this triad shapes the [Ca^2+^]_c_ signals and the exocytotic response in CCs (Montero et al. 2000; Garcia et al. 2006). If the location or the Ca^2+^‐handling properties of mitochondria in this functional triad could be regulated, this could become an effective mechanism for modulation of the exocytotic process. This Ca^2+^ handling by mitochondria could also modulate synaptic plasticity. Furthermore, under brain stress conditions such as excitotoxicity or ischemia‐reperfusion damage, aging or neurodegenerative diseases, mitochondrial dysfunction may reduce the ability of mitochondria to muffle cytosolic Ca^2+^, leading to increased secretion of excitatory neurotransmitters and cell overactivation, a vicious circle that may lead to Ca^2+^‐dependent neuronal death (Fernandez‐Morales et al. 2012).

In conclusion, our results provide the first functional features in support of the view that in BCCs there are two subpopulations of mitochondria, M1 underneath the plasmalemma nearby exocytotic sites and M2 at the core cell nearby vesicle transport sites. While M1 serves to shape the ACh‐elicited exocytotic response through its efficient Ca^2+^ removal by the mCUP, M2 shapes the lower [Ca^2+^]_c_ elevations required for new vesicles supply to the exocytotic machinery, from the large reserve vesicle pool at the cell core. The mCUP of the M1 pool seems to play a more prominent role in controlling the ACh response, in comparison with the mNCX.

## Conflict of Interest

None declared.
